# Engineering the MoS_2_/MXene Heterostructure for Precise and Noninvasive Diagnosis of Prostate Cancer with Clinical Specimens

**DOI:** 10.1002/advs.202206494

**Published:** 2023-03-29

**Authors:** Shaowei Xie, Xiaochen Fei, Jiayi Wang, Yi‐Cheng Zhu, Jiazhou Liu, Xinxing Du, Xuesong Liu, Liang Dong, Yinjie Zhu, Jiahua Pan, Baijun Dong, Jianjun Sha, Yu Luo, Wenshe Sun, Wei Xue

**Affiliations:** ^1^ Department of Urology Renji Hospital Shanghai Jiao Tong University School of Medicine Shanghai 200127 China; ^2^ Department of Ultrasound Renji Hospital Shanghai Jiao Tong University School of Medicine Shanghai 200127 China; ^3^ Central Laboratory Department of Ultrasound Pudong New Area People's Hospital Shanghai 201200 China; ^4^ Shanghai Engineering Research Center of Pharmaceutical Intelligent Equipment Shanghai Frontiers Science Research Center for Druggability of Cardiovascular Non‐coding RNA Institute for Frontier Medical Technology School of Chemistry and Chemical Engineering Shanghai University of Engineering Science Shanghai 201620 China; ^5^ Cancer Institute, The Affiliated Hospital of Qingdao University Qingdao 266071 China

**Keywords:** MoS_2_/MXene, noninvasive, prostate cancer, self‐assembly, urinary metabolic fingerprinting

## Abstract

High‐throughput metabolic fingerprinting has been deemed one of the most promising strategies for addressing the high false positive rate of prostate cancer (PCa) diagnosis in the prostate‐specific antigen (PSA) gray zone. However, the current metabolic fingerprinting remains challenging in achieving high‐precision metabolite detection in complex biological samples (e.g., serum and urine). Herein, a novel self‐assembly MoS_2_/MXene heterostructure nanocomposite with a tailored doping ratio of 10% is presented as a matrix for laser desorption ionization mass spectrometry analysis in clinical biosamples. Notably, owing to the two‐dimensional architecture and doping effect, MoS_2_/MXene demonstrates favorable laser desorption ionization performance with low adsorption energy, which is evidenced by efficient urinary metabolic fingerprinting with an enhanced area under curve (AUC) diagnosis capability of 0.959 relative to that of serum metabolic fingerprinting (AUC = 0.902) for the diagnosis of PCa in the PSA gray zone. Thus, this MoS_2_/MXene heterostructure is anticipated to offer a novel strategy to precisely and noninvasively diagnose PCa in the PSA gray zone.

## Graphical abstract

A novel self‐assembling MoS_2_/MXene heterostructure is synthesized as a matrix for laser desorption ionization mass spectrometry analysis to accurately diagnose prostate cancer in the prostate‐specific antigen (PSA) gray zone, resulting in a diagnostic capability of 0.959 (area under curve, AUC). Thus, MoS_2_/MXene heterostructure is expected to provide a novel approach to prostate cancer management in the PSAgray zone.

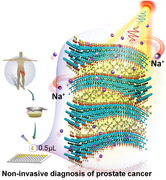

## 1 Introduction

Prostate cancer (PCa) is the most commonly diagnosed cancer and the leading cause of cancer death in men worldwide.^[^ ^1^, ^2^ ^]^ Early diagnosis and prompt radical prostatectomy are critical to improving the long‐term prognosis.^[^ ^3^, ^4^ ^]^ As the most widely used biomarker for PCa screening, the prostate‐specific antigen (PSA) provides limited sensitivity and specificity, which may lead to overdiagnosis and subsequent overtreatment.^[^ ^5^, ^6^, ^7^ ^]^ Especially, the high false positives (≈75%) in the PSA gray zone of 4–10 ng mL^−1^ lead to many unnecessary biopsies,^[^ ^8^, ^9^ ^]^ which aggravate the medical/physiological burden of patients and affect their life quality.^[^ ^8^, ^10^ ^]^ Meanwhile, imaging techniques such as ultrasound and magnetic resonance (MR) suffer from low accuracy, high costs, radiologist dependency, and time‐consuming testing, rendering them unsuitable for the practical, precise screening of PCa in the gray zone.^[^ ^11^, ^12^ ^]^ In this context, a precise and noninvasive platform is desirable for PCa diagnosis.

Targeted metabolites, as opposed to proteins, peptides, and genes, serve as direct signatures of biochemical reactions, which are intimately related to PCa occurrence.^[^ ^13^, ^14^ ^]^ Therefore, an efficient metabolic fingerprinting analysis may assist in the precise diagnosis of PCa. Mass spectrometry (MS) has been considered the gold standard for detecting metabolites owing to its high sensitivity and specificity, as well as its high‐throughput and ultrafast quantification.^[^ ^15^ ^]^ Notably, traditional metabolomics techniques such as liquid chromatography (LC) MS is limited by cumbersome pretreatment procedures.^[^ ^16^ ^]^ Alternatively, matrix‐assisted laser desorption/ionization (MALDI) MS, extensively applied in metabolic fingerprinting analyses, offers the advantages of ultrafast analysis, precise quantification, and a simple process.^[^ ^17^, ^18^ ^]^ The MALDI matrix is critical in metabolic fingerprinting analyses to realize highly efficient desorption/ionization and sensitive detection of targeted biomolecules (e.g., metabolites). A preferable matrix should efficiently adsorb and transfer energy from the laser to targeted analytes (e.g., metabolites), thereby enhancing the ionization process. Traditional organic matrices, such as the dihydroxybenzoic acid and *α*‐cyano‐4‐hydroxycinnamic acid, are unsuitable because of hot spots, inhomogeneous crystallization, and a high background signal, resulting in poor reproducibility and unsatisfactory capabilities.^[^ ^19^, ^20^ ^]^ Therefore, novel matrices that integrate the characteristics of feasible synthesis and favorable ionization efficiency are still urgently required.

MXenes belong to the family of atomically thin, two‐dimensional (2D) transition metal carbides and carbonitrides with intriguing properties,^[^ ^21^, ^22^, ^23^, ^24^, ^25^ ^]^ especially favorable efficiencies in ionization adsorption^[^ ^26^ ^]^ and energy transfer.^[^ ^27^, ^28^ ^]^ Molybdenum disulfide (MoS_2_) demonstrated higher efficiency in MALDI analysis than conventional nanomaterials owing to its high surface area and favorable thermal conductivity.^[^ ^29^, ^30^ ^]^ Furthermore, self‐assembly heterojunctions (e.g., MoS_2_‐based heterostructure) could reportedly further assist charge transfer and ionization,^[^ ^31^, ^32^ ^]^ which may make them an ideal matrix for MALDI‐based metabolic fingerprinting. Nevertheless, the synergistic performance of in situ self‐assembly MoS_2_/MXene heterostructure‐based matrices in MALDI‐MS has not been investigated. Thus, exploring whether such matrices can address the above limitations to facilitate advanced metabolic fingerprinting is crucial for the efficient diagnosis of PCa.

In this study, we proposed a novel LDI MS strategy by integrating MXene and MoS_2_ to self‐assemble, forming a matrix for direct serum/urinary metabolic fingerprinting (SMF/UMF) to precisely diagnose PCa in the PSA gray zone. The self‐assembly MoS_2_/MXene heterostructure matrix efficiently assisted the adsorption and transfer of energy from the laser to targeted analytes, resulting in enhanced LDI capability. The improved ionization efficiency was validated using theoretical density function theory (DFT) calculations, with a relatively low adsorption energy of 2.84 kcal mol^−1^. Furthermore, we used MoS_2_/MXene‐based SMF/UMF to differentiate PCa from benign prostatic hyperplasia (BPH) in the PSA gray zone. For SMF and UMF analyses, serum cohort (68 PCa versus 120 BPH) and urinary cohort (60 PCa versus 96 BPH) biosamples were collected for evaluation. The self‐assembly MoS_2_/MXene heterostructure enables efficient UMFfor PCa and BPH in the PSA gray zone, with an enhanced area under the curve (AUC) diagnosis capability of 0.959 relative to that of SMF (AUC = 0.902). This study sheds light on 2D heterostructure nanomaterials for efficient UMF capability, thus opening a novel avenue for precise and noninvasive diagnosis of PCa in the PSA gray zone.

## 2 Results and Discussion

### 2.1 Preparation and Characterizations of MoS_2_/MXene

**Figure 1:**
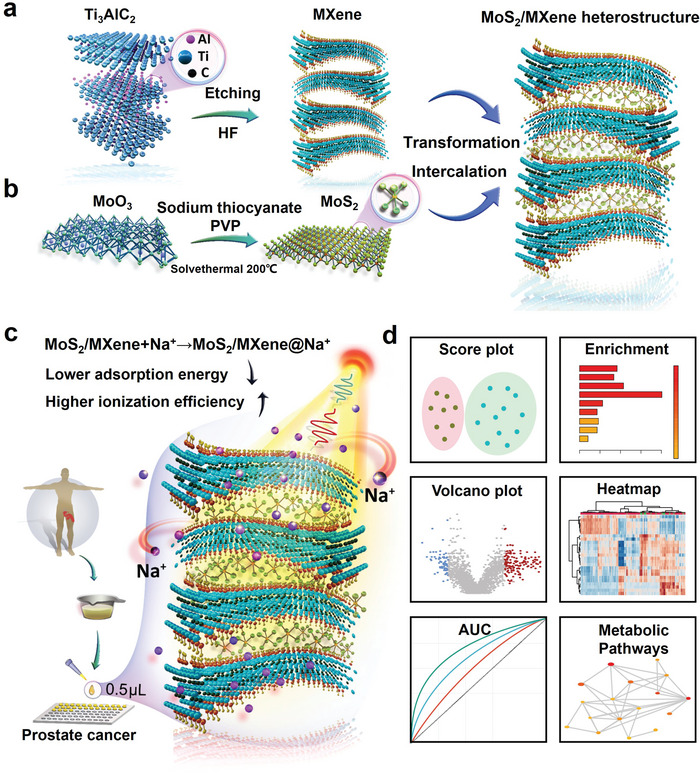
Schematic illustration of MoS_2_/MXene heterostructure‐based metabolic fingerprinting toward the efficient diagnosis of prostate cancer (PCa) in the prostate‐specific antigen (PSA) gray zone. a) Hydrofluoric acid (HF) etching of Ti_3_AlC_2_ in MXene. b) Solvothermal synthesis of MoS_2_ and in situ synthesis of MoS_2_/MXene heterojunction nanomaterial via hydrothermal reaction (120 °C, 20 h). c) Enhanced LDI analysis of small metabolites in clinical biosamples via MoS_2_/MXene with unique two‐dimensional (2D) architecture and doping effect. The MoS_2_/MXene heterojunction nanocomposites lowered the adsorption energy, enhancing the ionization efficiency. d) Score plot, enrichment overview, volcano plot, heatmap, area under curve (AUC), and metabolic pathways for accurate and noninvasive diagnosis of PCa.

In this study, tailored MoS_2_/MXene heterojunction nanocomposites were designed for metabolic fingerprinting of PCa. First, the multilayer 2D MXene nanocomposites were etched using hydrofluoric acid (HF) acid (**Figure** **1**a), and then, MoS_2_ particles were prepared at 200 °C via a solvothermal reaction of polyvinylpyrrolidone (PVP), sodium thiocyanate, and molybdenum oxide (Figure 1b).^[^ ^33^ ^]^ Subsequently, we performed the synthesis of MoS_2_/MXene heterojunction nanomaterial via hydrothermal reaction (120 °C, 20 h). Following MoS_2_/MXene synthesis, serum and urine samples were collected from patients with PCa and benign BPH in the PSA gray zone (Figure 1c). Finally, a comprehensive and in‐depth analysis was performed on metabolic fingerprinting towards the efficient diagnosis of PCa (Figure 1d), including a score plot, enrichment overview, volcano plot, heatmap, AUC, and metabolic pathway analysis, which may establish a novel avenue for precise and noninvasive diagnosis of PCa in the PSA gray zone.

**Figure 2:**
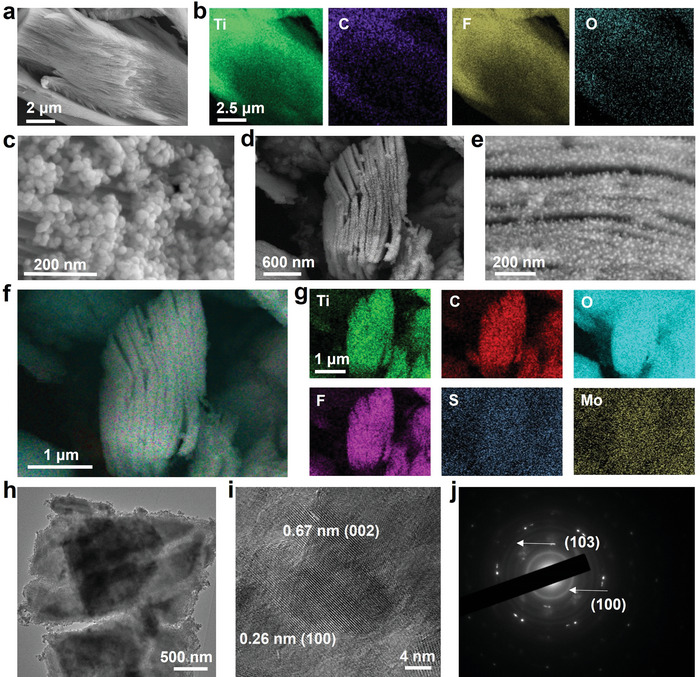
Characterizations of MoS_2_/MXene. a) Scanning electron microscopy (SEM) image and b) mapping analysis of MXene, including Ti, C, F, and O. c) SEM image of MoS_2_ nanoparticles. d) SEM image and e) magnified SEM image of MoS_2_/MXene heterostructure nanocomposites. MoS_2_ nanoparticles were uniformly distributed in the MXene intercalation structure. The unique distribution architecture of MoS_2_/MXene may improve the efficient ionization of small metabolic molecules. f) Merged mapping image and g) elemental mapping analysis of MoS_2_/MXene, including Ti, C, F, O, Mo, and S. h) Transmission electron microscopy (TEM) and i) high‐resolution TEM (HRTEM) image of MoS_2_/MXene. Obvious lattice fingerprints of 0.26 and 0.67 nm corresponded to the d‐spacing of the (100) plane of Ti_3_C_2_ and the (002) plane of MoS_2_, revealing that Ti_3_C_2_ and MoS_2_ were present in the heterostructure. j) Selected area electron diffraction (SAED) analysis of MoS_2_/MXene. The diffraction rings of Ti_3_C_2_ (103) and MoS_2_ (101) were detected.

The scanning electron microscopy (SEM) characterization of pristine MXene nanocomposites was depicted in **Figure** **2**a, showing a clear intercalation architecture. Moreover, the mapping analysis displayed the elemental distributions of the pristine MXene, in which C and Ti were structured, and F and O were distributed on the surface of the 2D MXene nanocomposites (Figure 2b and Figure S1, Supporting Information), demonstrating the successful synthesis of MXene. In addition, SEM showed that MoS_2_ nanoparticles had a particle size distribution of 26.4 ± 4.7 nm (Figure 2c). After synthesizing the MoS_2_/MXene heterostructure, the SEM photograph demonstrated that the MoS_2_ nanoparticles were uniformly distributed in the MXene intercalation architecture (Figure 2d,e). Furthermore, the merged and elemental mapping diagram revealed that Ti, C, O, F, S, and Mo were observed in the MoS_2_/MXene nanocomposites (Figure 2f,g, and Figure S2, Supporting Information), demonstrating the successful synthesis of MoS_2_/MXene heterostructure nanocomposites. Simultaneously, the UV–Vis spectral analysis (Figure S3, Supporting Information) of MoS_2_ and MXene indicated full absorption in the wavelength range of 420–900 nm due to the narrow band gap and nonplasmonic metallic structure, respectively, which were consistent with previous reports.^[^ ^34^, ^35^ ^]^ Meanwhile, for the structure‐properties correlation, we have conducted XRD characterizations for MoS_2_, MXene, and MoS_2_/MXene, characteristic peaks (29.02° and 39.83° for (004) and (002), respectively) validated the structures of our MoS_2_/MXene (Figure S4, Supporting Information).^[^ ^36^, ^37^ ^]^ Moreover, we performed finer morphological characterization and lattice spacing analysis via transmission electron microscopy (TEM) and high‐resolution TEM (HRTEM) for further verifications of the MoS_2_/MXene heterostructure nanocomposites (Figure 2h,i). The crystal nanostructures showed obvious lattice spacings of 0.26 and 0.67 nm, corresponding to the d‐spacing of the planes of Ti_3_C_2_ (103) and MoS_2_ (002) (Figure 2i), demonstrating the presence of Ti_3_C_2_ and MoS_2_ in the heterostructure.^[^ ^38^, ^39^ ^]^ Ultimately, we performed selected area electron diffraction (SAED) on the MoS_2_/MXene heterostructure and found the diffraction rings of Ti_3_C_2_ (103) and MoS_2_ (101),^[^ ^40^, ^41^ ^]^ again confirming the successful synthesis of the MoS_2_/MXene nanocomposites (Figure 2j). Notably, this unique distribution architecture of MoS_2_/MXene may be beneficial for the efficient ionization of small molecules, facilitating metabolic fingerprinting.

### 2.2 Optimizations and Verifications of MoS_2_/MXene

**Figure 3:**
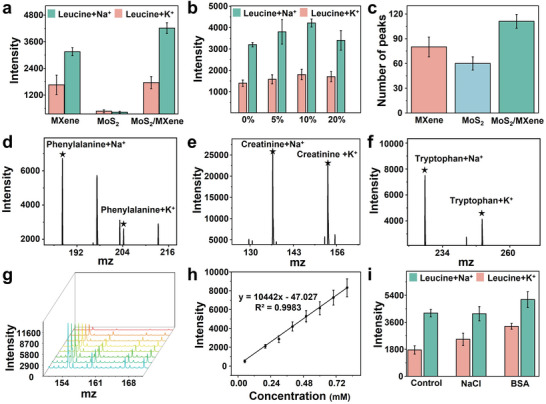
Optimizations and verifications of MoS_2_/MXene heterostructure nanocomposites for LDI analysis. a) Mass spectrometry (MS) analysis of leucine by pristine MXene, MoS_2_, and MoS_2_/MXene. b) Optimizations of the MoS_2_ to MXene ratio in MoS_2_/MXene. c) Peak count for biosample analysis using pristine MXene, MoS_2_ *_,_* and MoS_2_/MXene. d–f) Performances of optimized MoS_2_/MXene (doping ratio of 10%) for determinations of 1 ng nL^−1^ d) phenylalanine, e) creatinine, and f) tryptophan. g) LDI outcomes of MoS_2_/MXene for quantifying a series of leucine concentrations (0.05–0.8 × 10^−3^ m), and h) corresponding calibration curve. i) Salt tolerance and protein tolerance of MoS_2_/MXene for quantification of 1 ng nL^−1^ leucine in 0.5 m NaCl and 5 mg mL^−1^ bovine serum albumin (BSA).

To validate the LDI analysis of the MoS_2_/MXene nanocomposites, we used leucine as a standard molecule to optimize and verify the capability of MoS_2_/MXene in MS analysis. The MoS_2_/MXene demonstrated superior performances as compared to pristine MoS_2_ and MXene (**Figure** **3**a and Figure S5, Supporting Information). Moreover, tailored ratios of MoS2 and MXene were optimized. It was found that a ratio of 10% yielded optimal analytical outcomes (Figure 3b) and demonstrated the superior performance of LOD (limit of detection) and S/N (signal‐noise ratio) than commercial matrix for glucose detection (Table S1, Supporting Information) and acceptable reproducibility (Figure S6, Supporting Information). To further verify the clinical functionality, the number of MS peaks in the clinical serum biosamples was counted from three different matrices, and MoS_2_/MXene was found to exhibit the highest number of peaks (111 ± 8, Figure 3c, Figure S7, Supporting Information) and, indicating favorable clinical capability in metabolic fingerprinting. Furthermore, the MoS_2_/MXene (doping ratio at 10%) demonstrated favorable LDI capability for the detection of other standard molecules (10 ng nL^−1^), including phenylalanine, creatinine, and tryptophan, with distinct [M+Na]^+^ and [M+K]^+^ adducts (Figure 3d–f), verifying the universality of MoS_2_/MXene for metabolic detections. Moreover, the MoS_2_/MXene demonstrated favorable linearity for the quantification of leucine, ranging from 0.05 to 0.8 × 10^−3^ m (Figure 3g,h, *y* = 10442*x* – 47.027, *R* ^2^ = 0.9983), proving its quantitative capability for metabolic fingerprinting analysis. Finally, MoS_2_/MXene demonstrated favorable salt and protein tolerance to 0.5 m sodium chloride (NaCl) and 5 mg mL^−1^ bovine serum albumin (BSA), respectively (Figure 3i and Figure S8, Supporting Information). These validations confirmed the favorable feasibility of MoS_2_/MXene heterostructure nanocomposites toward LDI metabolic fingerprinting analysis.

### 2.3 DFT Calculations of MoS_2_/MXene Nanocomposites

**Figure 4:**
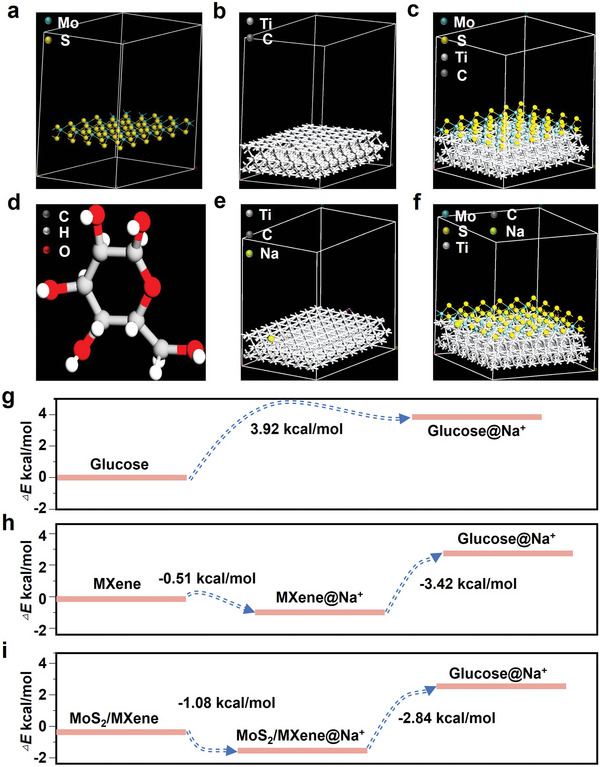
Density function theory (DFT) calculations of MoS_2_/MXene nanocomposites with higher ionization efficiency. a–f) Molecular models of a) MoS_2_, b) MXene, c) MoS_2_/MXene, d) glucose, e) MXene@Na^+^, and f) MoS_2_/MXene@Na^+^. g–i) Energy kinetics analysis of the ionization process for glucose g) without a matrix and with h) MXene, and i) MoS_2_/MXene. The ionization of glucose molecules without a matrix required 3.92 kcal mol^−1^ of energy (*E*_ad‐1_, Equation (1)), whereas it required 3.42 kcal mol^−1^ with MXene as the matrix (*E*_ad‐2_, Equation (2)). Notably, glucose with MoS_2_/MXene as the matrix only consumed 2.84 kcal mol^−1^ energy (*E*_ad‐3_, Equation (3)), demonstrating that MoS_2_/MXene effectively reduced the adsorption energy between glucose and Na^+^.

Investigating the intrinsic mechanism of a matrix is critical for practical applications of LDI metabolic fingerprinting analyses. Herein, ionization calculations were performed on matrices (MXene and MoS_2_/MXene) via DFT to investigate the LDI enhancement mechanism. Specifically, appropriate adsorption energy between glucose and Na^+^ is an indispensable procedure for the metabolic fingerprinting of metabolites.^[^ ^17^, ^18^ ^]^ The molecular models of MoS_2_, MXene, and MoS_2_/MXene were constructed using the Materials Studio 2019 software (**Figure** **4**a–c). Glucose was used as a typical metabolic molecule (Figure 4d and Figure S9, Supporting Information) to simultaneously investigate the ionization processes of MXene@Na^+^ and MoS_2_/MXene@Na^+^ (Figure 4e,f). The ionization of glucose molecules without matrix required 3.92 kcal mol^−1^ of energy (*E*_ad‐1_, Equation (1), Figure 4g), while it required 3.42 kcal mol^−1^ with MXene as the matrix (*E*_ad‐2_, Equation (2), Figure 4h). Notably, glucose with MoS_2_/MXene as the matrix consumed only 2.84 kcal mol^−1^ energy (*E*_ad‐3_, Equation (3), Figure 4i), demonstrating that MoS_2_/MXene effectively reduced the adsorption energy between glucose and Na^+^. Therefore, our molecular simulations revealed that MoS_2_/MXene effectively reduced the adsorption energy between metabolic molecules (e.g., glucose) with Na^+^, further enhancing the ionization process and improving LDI performances.

### 2.4 SMF/UMF of PCa and BPH via MoS_2_/MXene Heterostructure

**Figure 5:**
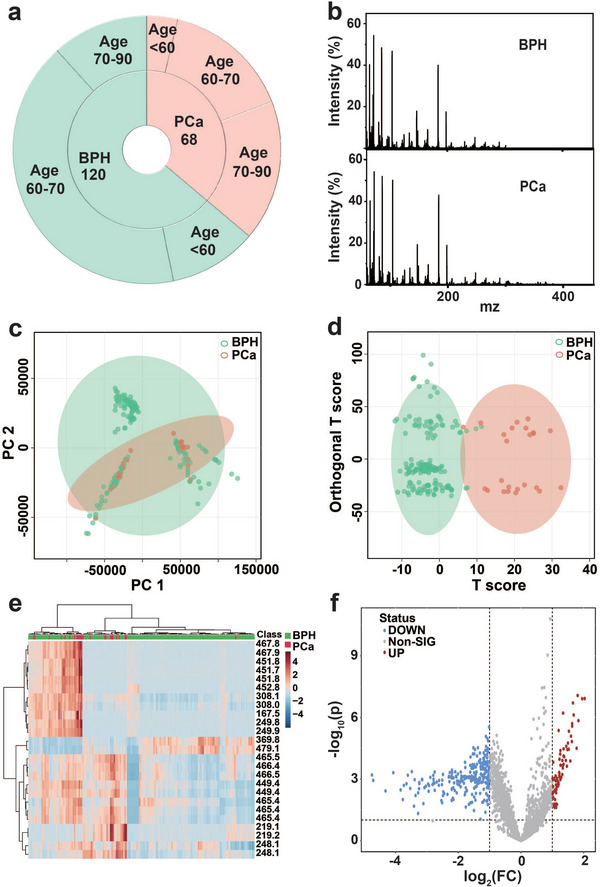
The serum metabolic fingerprinting (SMF) analyses of prostate cancer (PCa) and benign prostatic hyperplasia (BPH) via MoS_2_/MXene heterostructure. a) Clinical data of serum biosamples, including 68 PCa and 120 BPH biosamples. b) Representative mass spectrometry (MS) photographs of BPH and PCa. c) Principal component analysis (PCA) and d) orthogonal partial least squares discriminant analysis (OPLS‐DA) analysis of PCa and BPH. The OPLS‐DA strategy successfully discriminated PCa from BPH. e) Typical heatmap analysis presents evident metabolic differences (components and concentrations) between the two clinical cohorts. f) A volcano plot was utilized to demonstrate a series of upregulated and downregulated MZ (mass‐to‐charge ratio) panels (*p* < 0.05). The *x*‐ and *y*‐axes represent log_2_ (fold change) and −log_10_ (*p*‐value), respectively.

To validate the clinical feasibility of MoS_2_/MXene heterostructure nanocomposites, PCa, and BPH in the PSA gray zone were selected for SMF and UMF. We performed a Venn diagram (Figure S10, Supporting Information) analysis to show the homology of the urine and serum samples, which demonstrated the feasibility of combined diagnosis of blood/urine biosamples. For SMF, we included 68 PCa serum and 120 BPH serum biosamples (**Figure** **5**a), and all relevant clinical information for biosamples was described in Table S2 (Supporting Information). The typical mass spectra of BPH and PCa were presented in Figure 5b. To distinguish PCa from BPH, we further performed a classical unsupervised principal component analysis (PCA, Figure 5c) and supervised orthogonal partial least squares discriminant analysis (OPLS‐DA, Figure 5d). The PCA overlapped some samples and OPLS‐DA separated all samples with effective discrimination. Furthermore, a heatmap analysis was performed to select potential MZ (mass‐to‐charge ratio) as the biomarker panel. Heatmap analysis showed a series of MZ panels, demonstrating obvious metabolic differences (components and concentrations) between the two clinical cohorts (Figure 5e). Moreover, a volcano plot was used to visualize the *p‐*value and fold change value, which is beneficial for screening differential metabolites. The *x*‐axis represents log_2_ (fold change), and the *y*‐axis represents log_10_ (*p‐*value). The volcano map revealed up‐ and downregulated metabolites (Figure 5f and Figure S11, Supporting Information, *p* < 0.05), illustrating highly significant metabolites having low *p* values.

**Figure 6:**
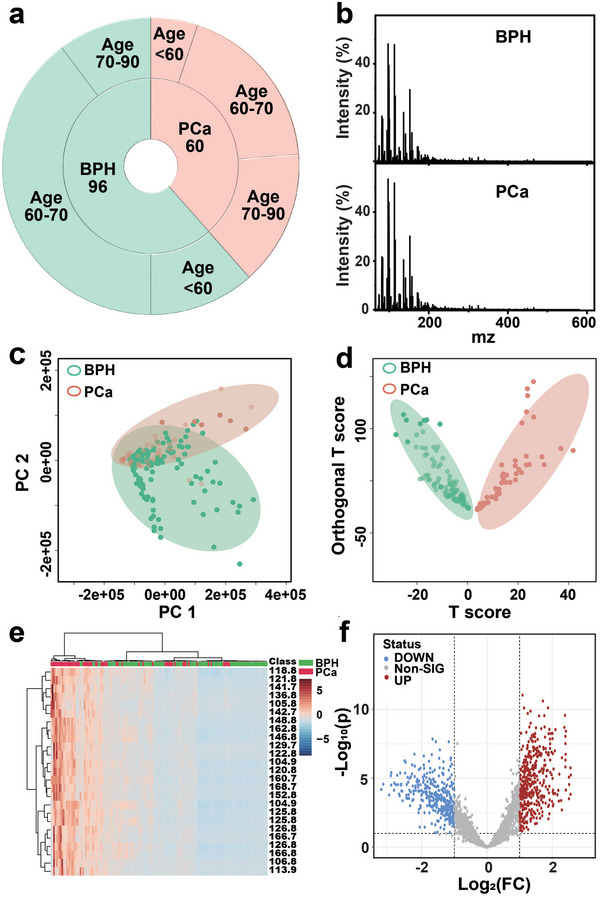
The urinary metabolic fingerprinting (UMF) of prostate cancer (PCa) and benign prostatic hyperplasia (BPH) via MoS_2_/MXene heterostructure. a) Clinical data of patients from whom urine biosamples were obtained, including 60 PCa and 96 BPH biosamples. b) Representative LDI analysis of BPH and PCa. c) Principal component analysis (PCA) and d) orthogonal partial least squares discriminant analysis (OPLS‐DA) analysis of PCa and BPH. The OPLS‐DA successfully discriminated PCa from BPH. e) Typical heatmap and f) volcano plot analyses were performed to identify a series of upregulated and downregulated MZ (mass‐to‐charge ratio) panels for subsequent metabolic pathway analysis.

**Figure 7:**
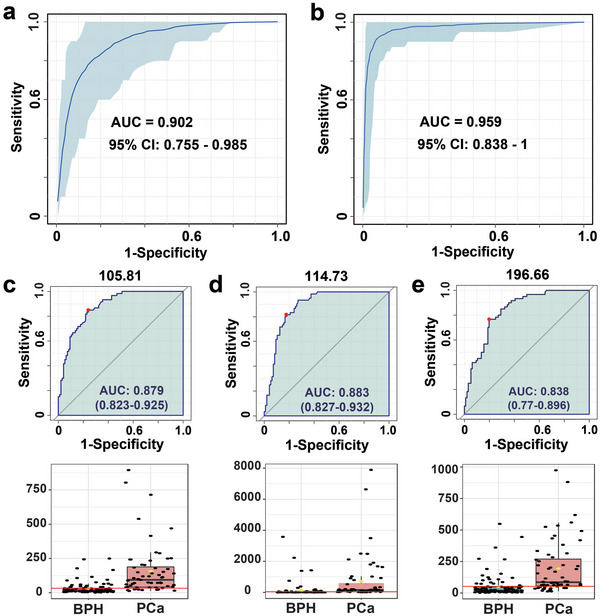
Comparisons of serum metabolic fingerprinting (SMF) and urinary metabolic fingerprinting (UMF) and targeted MZ (mass‐to‐charge ratio) quantitative analysis. a,b) Area under curve (AUC) values of a) SMF and b) UMF via the MoS_2_/MXene heterostructure for discriminating prostate cancer (PCa) from benign prostatic hyperplasia (BPH). UMF via MoS_2_/MXene was achieved with a superior AUC capability of 0.959 compared to SMF (AUC = 0.902). c–e) Urinary AUC values and statistical analysis of c) MZ = 105.81, d) MZ = 114.73, and e) MZ = 196.66 to discriminate PCa from BPH. Two clinical cohorts of PCa and BPH exhibited obvious metabolic differences with corresponding AUC values of 0.879, 0.883, and 0.838, respectively.

Considering the unique relationship between PCa and urine biosamples,^[^ ^42^, ^43^ ^]^ urine is a noninvasive biofluid with unique advantages in detection scenarios. We thus performed MoS_2_/MXene‐based UMF analysis on urinary biosamples from 60 PCa and 96 BPH patients (**Figure** **6**a) and all relevant clinical information for biosamples was described in Table S2 (Supporting Information). Typical LDI MS spectra of BPH and PCa were displayed in Figure 6b. Similarly, compared with PCA, OPLS‐SA also achieved successful discrimination of PCa from the BPH (Figure 6c,d). Furthermore, we plotted a heatmap (Figure 6e) and a volcano plot (Figure 6f), exhibiting a series of upregulated and downregulated MZ panels with significant fold changes (Figure S12, Supporting Information, *p* < 0.05). The SMF and UMF successfully distinguished PCa from BPH, and the AUC was further used to evaluate the diagnostic performances (**Figure** **7**a,b). The results revealed that UMF via MoS_2_/MXene demonstrated a superior AUC capability of 0.959 as compared to SMF (AUC = 0.902), which demonstrated the enhanced performance of UMF with the merit of being noninvasive in the accurate diagnosis of PCa in the PSA gray zone. Notably, we compared the performance of MoS_2_/MXene‐based UMF and SMF with clinical standard technologies including PSA and MR imaging in Table S3 (Supporting Information), demonstrating superior diagnostic capability for PCa management. Furthermore, for UMF, the two clinical cohorts of PCa and BPH revealed evident metabolic significance in MZ = 105.81, 114.73, and 196.66, with corresponding AUC values of 0.879, 0.883, and 0.838, respectively (Figure 7c–e). Significant MZ panels may serve as novel critical biomarkers to interpret the underlying mechanism of PCa in the PSA gray zone.

Disordered metabolic pathways are closely related to the occurrence of PCa.^[^ ^44^, ^45^ ^]^ Based on the selected MZ panel feature heatmaps and volcano plots with high refractive index changes and *p* values in UMF analysis, MZ metabolites were identified and searched using metabolic databases (e.g., HMDB, MetFrag, Massbank, and Metlin). Through enrichment pathway analysis (https://www.metaboanalyst.ca/home.xhtml), important pathways, including cysteine metabolism, homocysteine metabolism, pyruvaldehyde metabolism, and taurine metabolism, which may reveal the occurrence of PCa in the PSA gray zone, were identified (Figure S13a, Supporting Information). These metabolic pathways form an interactive network (Figure S13b, Supporting Information). The high metabolic activity of PCa produces a large number of reactive oxygen species,^[^ ^46^, ^47^ ^]^ which requires increased consumption of poly‐cysteine to maintain the redox balance.^[^ ^48^ ^]^ Therefore, the cysteine pathway may be activated to generate large amounts of antioxidants. It has also been reported that homocysteine metabolism is involved in the regional hypomethylation of DNA sequences, which is common in the early stages of tumorigenesis.^[^ ^49^ ^]^ Taurine has been reported to have a significant effect on PSA levels, which may involve a correlation between taurine metabolism, PSA, and PCa occurrence.^[^ ^50^ ^]^ In addition, taurine is involved in regulating sugar metabolism and accelerates glycolysis,^[^ ^51^, ^52^ ^]^ as confirmed by the metabolic enrichment of pyruvate (Figure S13a, Supporting Information). Aerobic glycolysis is a prominent feature of tumor cells,^[^ ^53^ ^]^ and PCa likely accelerates the rate of glycolysis via taurine to meet the needs for energy and biomaterial synthesis, thereby exhibiting proliferative and metastatic advantages and providing a potential target for intervention. Therefore, the network associations among all relevant metabolic pathways may provide useful guidance for exploring the mechanism underlying PCa occurrence in the PSA gray zone.

## 3 Conclusions

In summary, self‐assembly MoS_2_/MXene heterostructure nanocomposites were synthesized via a hydrothermal reaction strategy toward the accurate and noninvasive diagnosis of PCa in the PSA gray zone. The MoS_2_/MXene as the LDI matrix was systematically characterized, including 2D morphology, elemental compositions, and SEAD, and optimized with a tailored doping ratio of 10%, demonstrating favorable capability towards metabolic fingerprinting of standard molecules in serum and urine biospecimens. Notably, MoS_2_/MXene demonstrated favorable LDI performances, with lower adsorption energy because of the unique 2D architecture and doping effect, as verified by DFT calculations. Ultimately, for distinguishing PCa from BPH in the PSA gray zone, self‐assembly MoS_2_/MXene heterostructure enables efficient UMFwith an enhanced AUC diagnosis capability of 0.959 relative to that of SMF(AUC = 0.902), which may pave a novel avenue for accurate and noninvasive diagnosis of PCa in the PSA gray zone. Our work not only provides a versatile strategy for the design of a heterostructure‐based LDI matrix, but also paves the avenue for efficient cancer diagnosis and classification, or even identification of cancer biomarkers.

## 4 Experimental Section

### Chemicals and Materials

Molybdenum oxide (MoO_3,_ 99%), PVP (MW = 360000_,_ 99%), sodium sulfocyanate (NASCN_,_ 99%), NaCl, and HF were bought from Sinopharm Co., Ltd (Beijing, China). Standard molecules, including valine, phenylalanine, leucine, tryptophan, creatinine, and glucose, were obtained from Aladdin Reagent Co., Ltd (Shanghai, China). BSA was provided by Sigma‐Aldrich (St. Louis, Mo, USA).

### Synthesis of MoS_2_

To synthesize MoS_2_ nanoparticles, 10 mg MoO_3_ was added to 1 mL of a purified water solution containing 12 mg PVP.^[^ ^54^ ^]^ The mixture was magnetically stirred for 15 min to yield a heterogeneous, well‐distributed suspension. Substantially, 25 mg NASCN was added with vigorous stirring for 20 min. Afterward, the mixture was transferred to a Teflon stainless autoclave and heated at 200 °C for 36 h. The resulting product was centrifuged and cleaned three times with deionized water and ethanol, respectively. Lastly, the MoS_2_ nanoparticles were dried at 75 °C and stored in a vacuum environment until further use.

### Synthesis of MXene and Subsequent MoS_2_/MXene Heterojunction

MAX Ti_3_AlC_2_ was prepared to synthesize MXene.^[^ ^41^ ^]^ Ti was prepared via high‐temperature annealing of TiC, Al, and Ti powders at an atomic ratio of 2:1.5:1. Samples were heated to 1400 °C for 3 h. The heating and cooling rate was 10 °C min^−1^. Following annealing, the resulting Ti_3_AlC_2_ products were milled via a 100‐mesh sieve, yielding powders with an average diameter of fewer than 50 µm. For the chemical etching of Ti_3_AlC_2_, the MXene was synthesized via the selective etching of aluminum in Ti_3_AlC_2_ via 30% HF. The etching process lasted 10 h at room temperature. Thereafter, the solution was centrifuged and rinsed with deionized water thrice until the pH reached 4.9. Then, the MXene nanocomposites were dried in a vacuum for further use. For the next transformation of the MoS_2_/MXene heterojunction, different ratios of MoS_2_ and MXene were dispersed in 1 mL of deionized water under ultrasonication. Then, the mixture was transferred into a Teflon autoclave and heated at 160 °C for 5 h, resulting in a gray powder (denoted as MoS_2_/MXene, after ethanol/water washing and drying) for further applications.

### Characterizations and Apparatus

Morphological characterizations were performed using SEM (Hitachi S‐4800S, Tokyo, Japan) and TEM (JEM 2100F, Tokyo, Japan). SAED of MoS_2_/MXene was characterized using JEM 2100F (JEOJ, Tokyo, Japan). A UV 752 (Juchuang Environmental Co., Ltd., China) instrument was used to characterize the UV–Vis spectra of the matrices.

### MoS_2_/MXene‐Assisted LDI MS Analysis

All LDI MS analyses were conducted on a MetaDx MS (Tailai Biosciences Co, China), equipped with a 355‐nm ND: YAG laser beam for MS fingerprinting. The obtained LDI MS spectra were automatically analyzed using the flexAnalysis software (Diagno MS‐TXT). For a typical quantification procedure, 0.5 µL of targeted biosamples (e.g., urine or serum) was spotted on a 96‐polished steel plate. Subsequently, the biosamples were air‐dried before being mixed with a 0.5 µL matrix (e.g., MXene and MoS_2_/MXene, 1.0 mg mL^−1^). All LDI MS spectra were acquired in the linear mode with a laser frequency of 60 Hz.^[^ ^55^ ^]^ For the detailed configurations, a random walk of 40 or 30 different spots was calculated. A mixture of phenylalanine, leucine, and tryptophan was dissolved in deionized water with a concentration of 10 ng nL^−1^ to verify the universality and quantitative capability of MoS_2_/MXene for metabolic detection. To evaluate and compare salt and protein tolerance, 0.5 m NaCl and 5 mg mL^−1^ BSA were added to a mixture of standard molecules (10 ng nL^−1^).

### Collection of Clinical Serum and Urine Biosamples

For collecting urine biosamples, 15 mL of urine was centrifuged at 3000 × *g* for 10 min and stored at −80 °C. To collect serum biosamples, 5 mL of collected venous blood was centrifuged at 3000 × *g* for 5 min and stored at −80 °C.^[^ ^55^ ^]^ All study protocols were approved by the Ethics Committee of Renji Hospital, Shanghai Jiaotong University School of Medicine (KY2021‐030). Signed informed consent was obtained from all participants.

### DFT Stimulation of MoS_2_/MXene

All DFT calculations were performed using Materials Studio 2019 with the Doml^3^ software.^[^ ^56^, ^57^ ^]^ The pristine cell of disulfide molybdenum was imported from the standard software procedures, including the 2 × 2 × 2 crystal. The vacuum distance was set at 20 Å. A generalized gradient approximation was selected for calculation.^[^ ^57^ ^]^ The following configurations were chosen for the geometry optimizations: convergence standards of 10^−5^ Ha on energy, 2 × 10^−3^ Ha Å^−1^ on the force, and 5 × 10^−3^ Å on displacement. The adsorption energy (*E*_ads_) was used to evaluate the strength of the ionization interaction between small metabolites and Na^+^. The *E*_ads_ values for the ionization processes for control, pristine MXene, and MoS_2_/MXene were defined as *E*_ads‐1_, *E*_ads‐2_, and *E*_ads‐3_, respectively.

(1) $$E_{\mathrm{ads}-1}=E_{{\left[\mathrm{Glucose}@\mathrm{Na}\right]}^{+}}\;-E_{\mathrm{Glucose}}-E_{Na^{+}}$$

(2) $$E_{\mathrm{ads}-2}=E_{\mathrm{MXene}}+E_{{\left[\mathrm{Glucose}@\mathrm{Na}\right]}^{+}}\;-E_{\mathrm{Glucose}}-E_{{\left[\mathrm{MXene}@\mathrm{Na}\right]}^{+}}\;$$

(3) $$\begin{array}{ccc} E_{\mathrm{ads}-3} & = & E_{\mathrm{MoS}2/\mathrm{MXene}}+E_{{\left[\mathrm{Glucose}@\mathrm{Na}\right]}^{+}}\;-E_{\mathrm{Glucose}}\\ & & -\;E_{{\left[\mathrm{MoS}2/\mathrm{MXene}@\mathrm{Na}\right]}^{+}} \end{array}$$

### Identification of Metabolites and Metabolic Pathway Analysis

The metabolite MZ identification was carried out using the following metabolic databases: Human Metabolome Database (HMDB, https://hmdb.ca/spectra/ms/search), MetFrag (Waters, https://ipb‐halle.github.io/MetFrag), Metlin (https://metlin.scripps.edu/), and Massbank (http://www.massbank.jp/). All metabolic analyses, such as PCA and partial least squares discriminant analysis, were performed using the user‐friendly, streamlined metabolomics data analysis tool MetaboAnalyst 5.0 (https://www.metaboanalyst.ca/home.xhtml).^[^ ^58^ ^]^ This online software also helps with the volcano plot, heatmap, enrichment analysis, and metabolic pathway analysis. Each MZ feature with a fold change >1.5 and a *t*‐test threshold *p*‐value < 0.05 was in the volcano plots. All data are presented as the mean ± SD (standard deviation) and were obtained using the GraphPad Prism 5.01 statistical software.^[^ ^59^ ^]^

## Conflict of Interest

The authors declare no conflict of interest.

## Author Contributions

S.X. investigated, conceptualized, supervised, wrote, and edited the work. X.F. investigated and also worked on data curation. J.W. validated the work. Y‐C.Z. worked on project administration. J.L. performed biosample collection. X.D. performed biosample collection. X.L. performed formal analysis and L.D. worked on project administration. Y.Z. validated and J.P. reviewed and edited the work. B.D. reviewed and edited the work. J.S. supervised the work. Y.L. supervised and reviewed the work. W.S. supervised and reviewed the work. W.X. conceptualized, supervised, reviewed, wrote, and edited the work.

## Supporting information

Supporting Information

## Data Availability

The data that support the findings of this study are available on request from the corresponding author. The data are not publicly available due to privacy or ethical restrictions.
