# Supplementary material for: Engineering the MoS2/MXene Heterostructure for Precise and Noninvasive Diagnosis of Prostate Cancer with Clinical Specimens
Source: Adv Sci (Weinh). 2023 Mar 29;10(15):2206494. doi: 10.1002/advs.202206494 (PMC10214233; doi:10.1002/advs.202206494)
Supplement: Supplementary file 1 — Supporting Information [file ADVS-10-2206494-s001.pdf]

ADVANCED SCIENCE
Open Access

Supporting Information

for *Adv. Sci.*, DOI 10.1002/advs.202206494

Engineering the MoS_2/MXene Heterostructure for Precise and Noninvasive Diagnosis of Prostate Cancer with Clinical Specimens

Shaowei Xie, Xiaochen Fei, Jiayi Wang, Yi-Cheng Zhu, Jiazhou Liu, Xinxing Du, Xuesong Liu, Liang Dong, Yinjie Zhu, Jiahua Pan, Baijun Dong, Jianjun Sha, Yu Luo, Wenshe Sun* and Wei Xue**

Engineering the MoS_2/MXene Heterostructure for Precise and Non-invasive Diagnosis of Prostate Cancer with Clinical Specimens

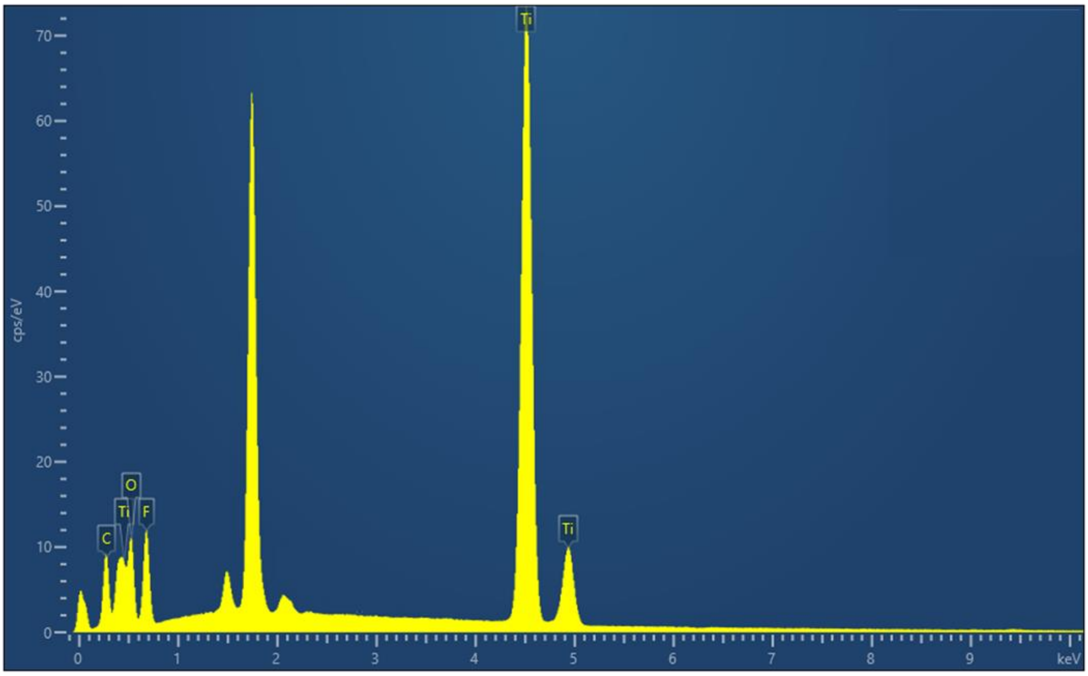

Fig. S1. The EDS analysis of MXene. C, Ti, C, F and O were detected, which evidenced the successful synthesis of MXene.

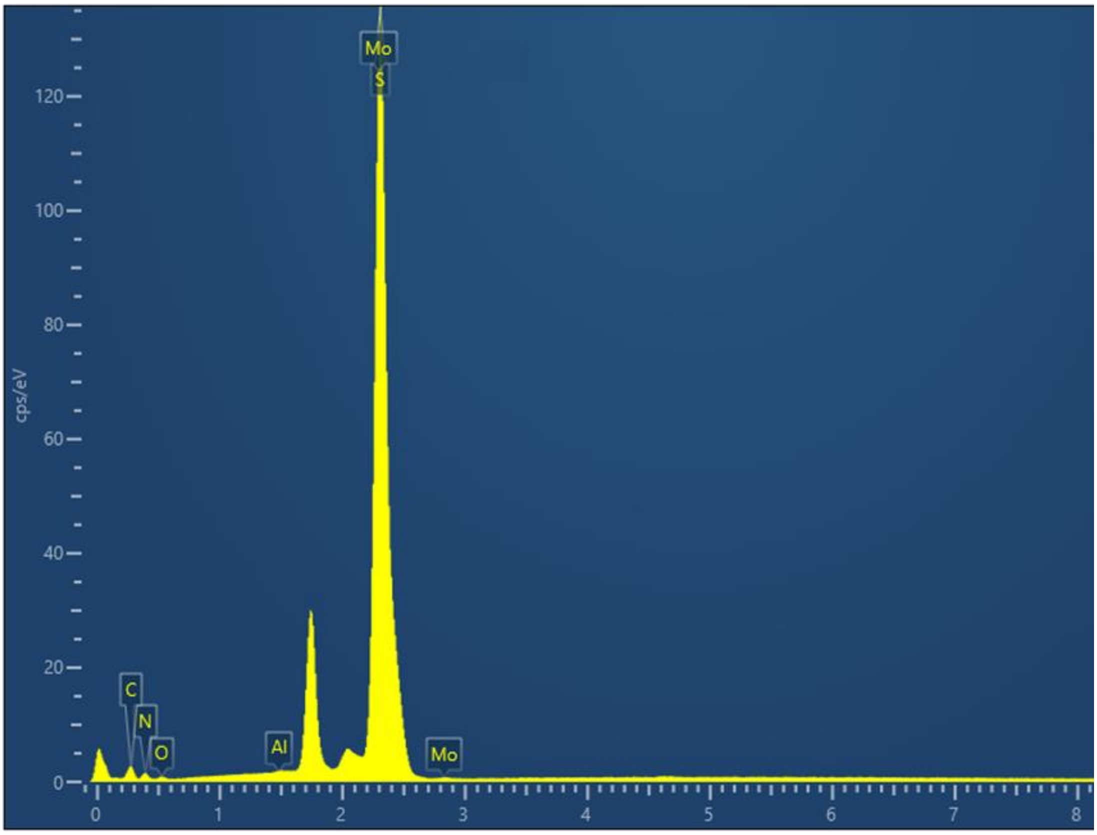

Fig. S2. EDS analysis of MoS_2/MXene. Extra elemental existence including Mo and S validated the successful synthesis of MoS_2/MXene heterostructure nanocomposites.

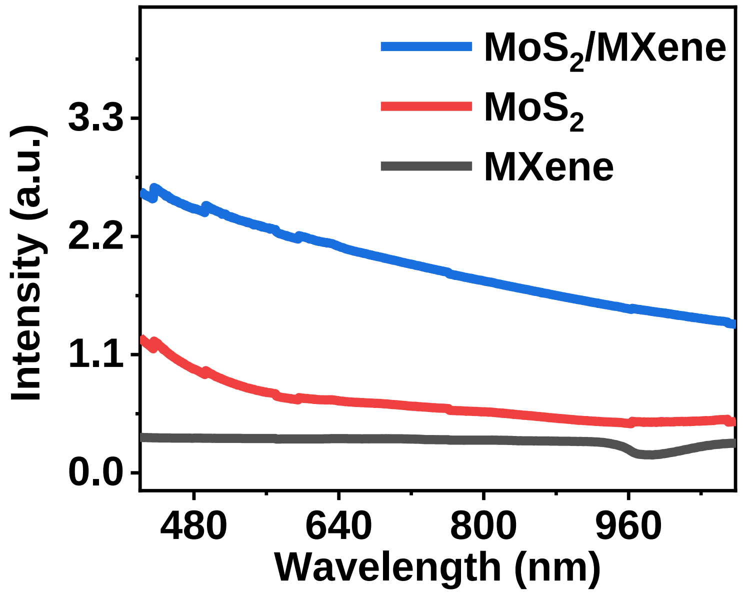

Fig. S3. UV-vis analysis of MoS_2, MXene, and MoS_2/MXene. The spectra of MoS_2 and MXene indicated that there is full absorption in the 420-900 nm range due to the narrow band gap, black color and non-plasmonic metallic structure, respectively.

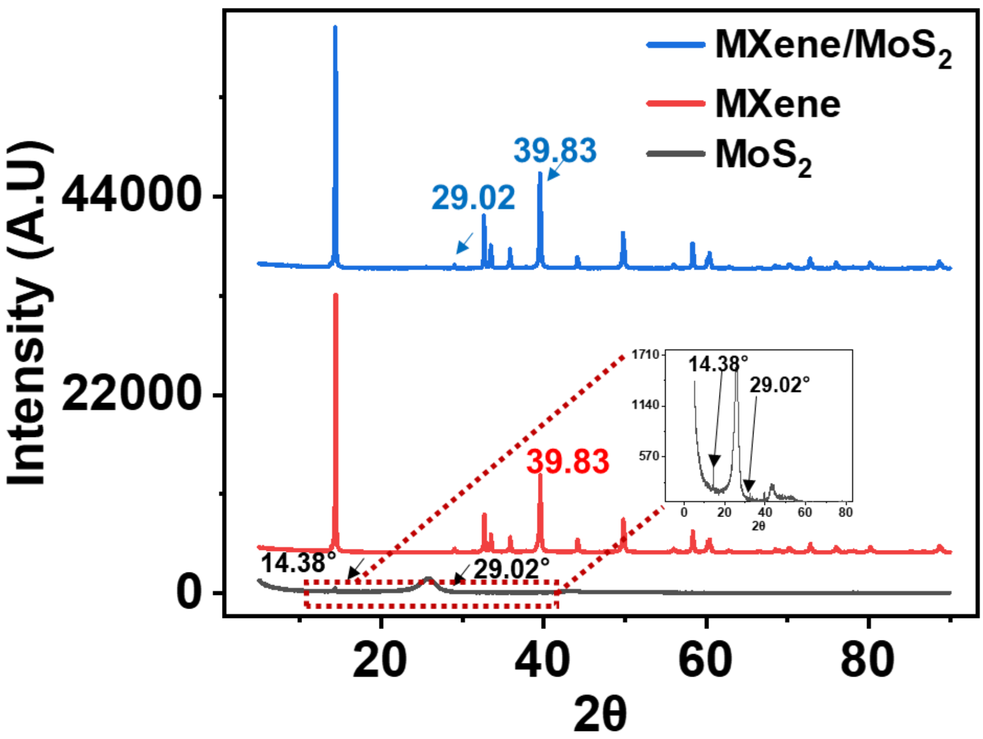

Fig. S4. XRD characterizations for MoS_2, MXene, and MoS_2/MXene, characteristic peaks (29.02° and 39.83° for (004) and (002), respectively) validated the structures of our MoS_2/MXene.

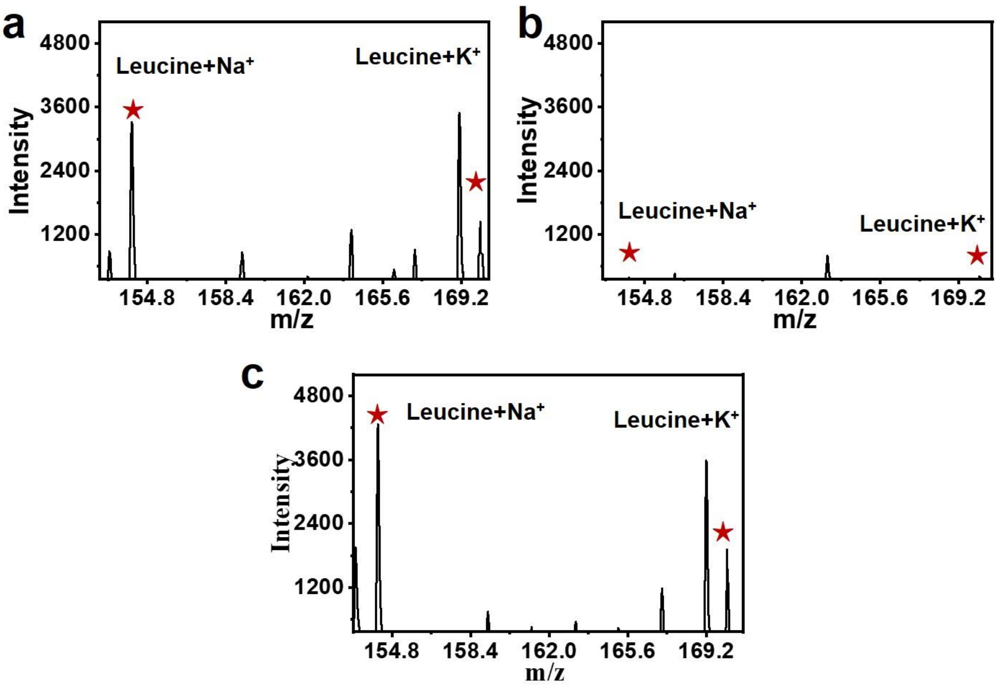

Fig. S5. Selections of matrices for LDI MS fingerprinting. Performance of a) MXene, b) MoS_2, c) MoS_2/MXene for determination of 1 ng/nL leucine standard molecule. The $[M+Na]^+$ and $[M+K]^+$ adducts were selected for characterizations.

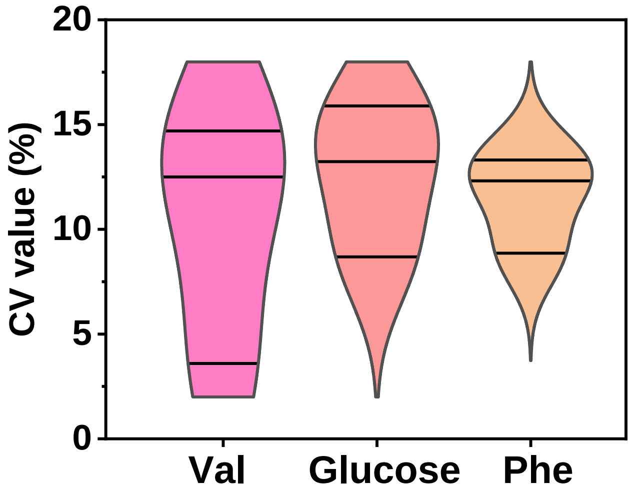

Fig. S6. CV (coefficient of variation) analysis of MoS_2/MXene for detection of valine, glucose, and phenylalanine, demonstrating favorable reproducibility of MoS_2/MXene.

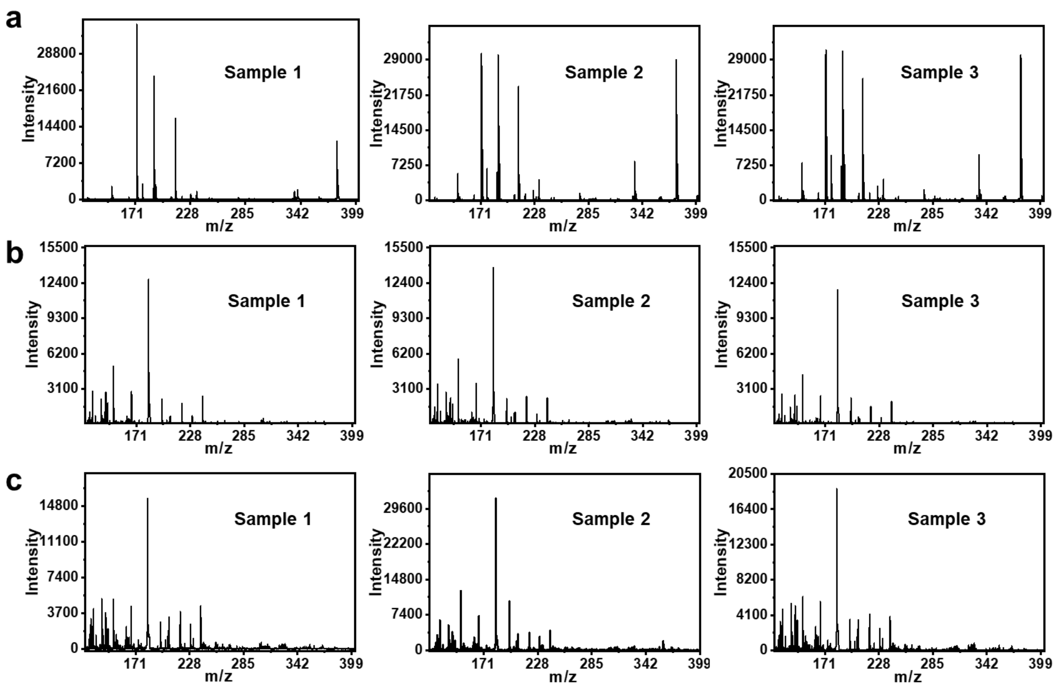

Fig. S7. Optimizations of MoS_2/MXene towards clinical performance. MS spectra of serum biosamples (n = 3) via a) MXene, b) MoS_2, c) MoS_2/MXene. The number of peaks of each matrix were calculated and compared with the signal noise ratio (S/N) of 3.0.

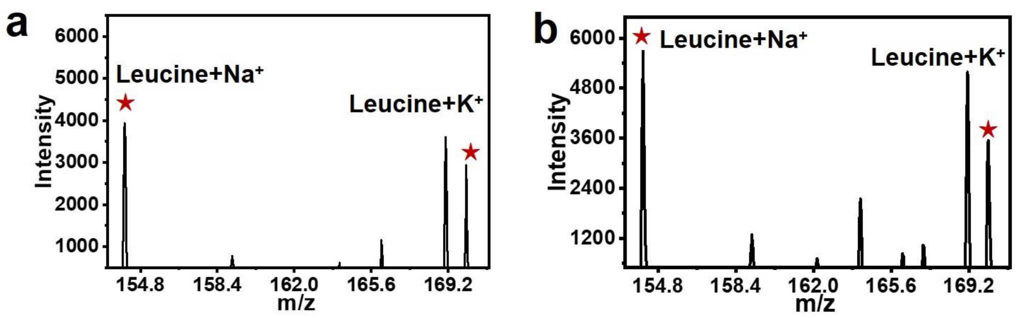

Fig. S8. a) Salt and b) protein tolerance of MoS_2/MXene heterostructure nanocomposites. The average intensity of $[Leucine+Na]^+$ and $[Leucine+K]^+$ by using MoS_2/MXene in 0.5 M NaCl and 5 mg/mL BSA were calculated for validations.

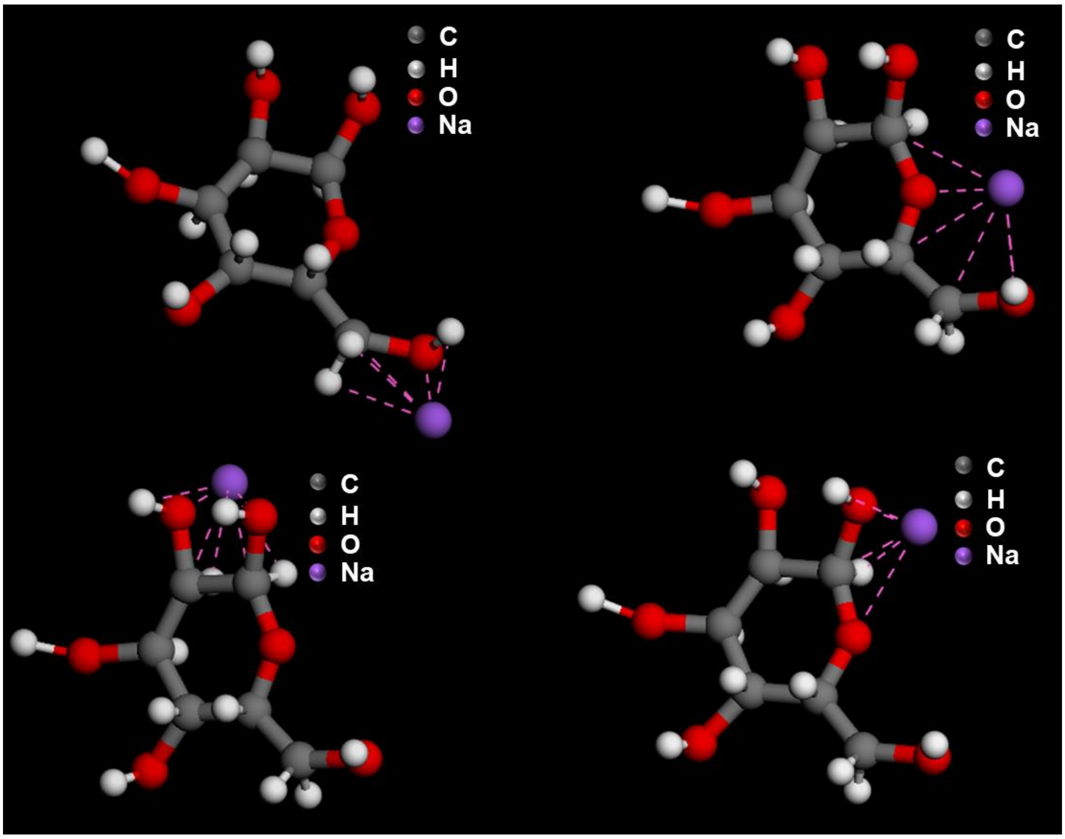

Fig. S9. Molecular structures of [Glucose@Na]$^{+}$.

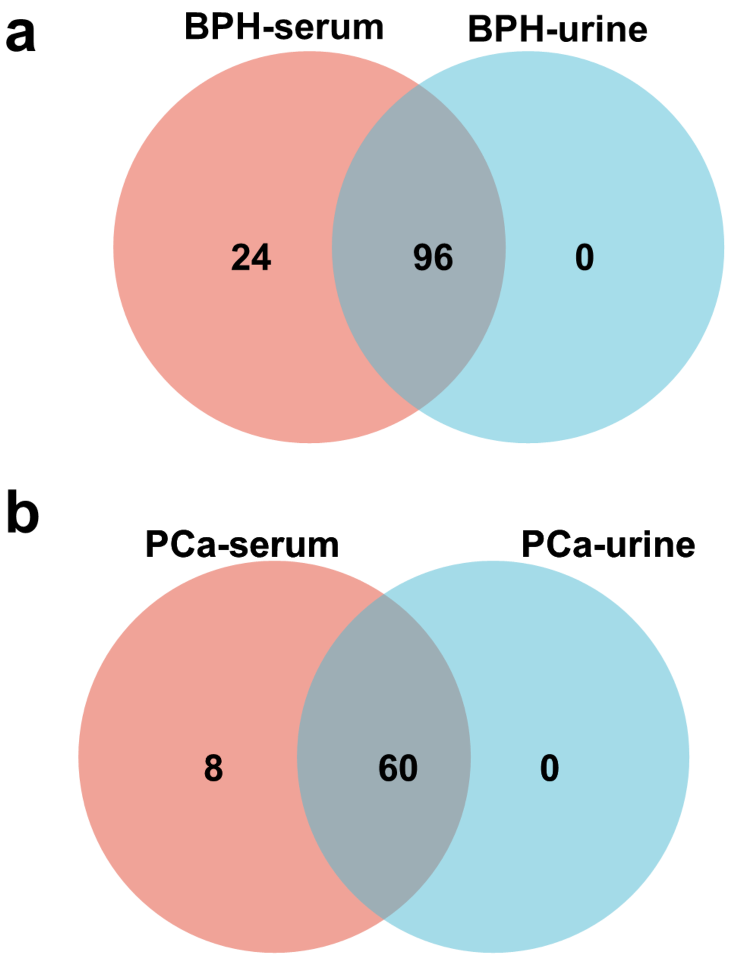

Fig. S10. Venn diagram of serum and urine biosamples demonstrated the homology of biospecimens.

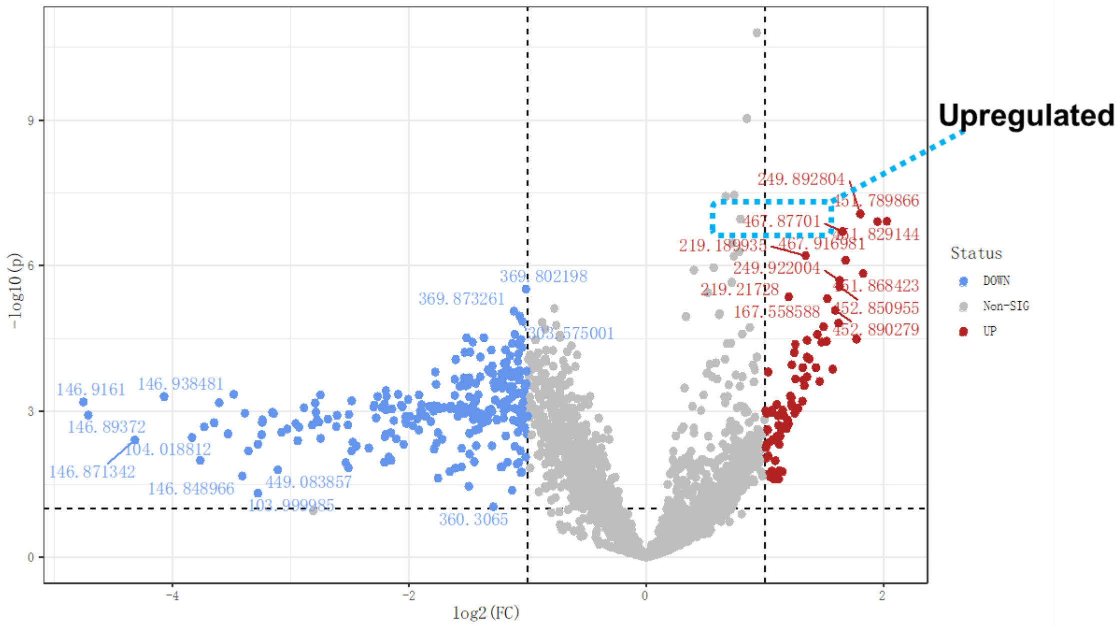

Fig. S11. Volcano plot of serum metabolites with labeled upregulated MZ panel. The x-axis was represented by $\log_2$ (fold change), and the y-axis was represented by $-\log_{10}$ (*p*-value). The red dots represented the upregulated MZ panel and the blue dots was the downregulated MZ panel.

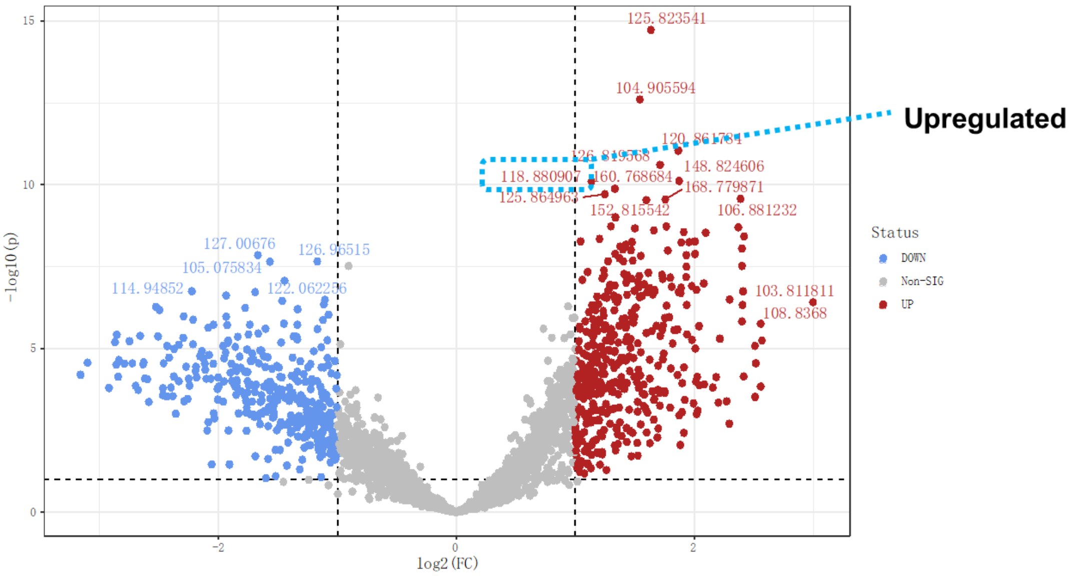

Fig. S12. Volcano plot of urinary metabolites with labeled upregulated MZ panel. The x-axis was represented by $\log_2$ (fold change), and the y-axis was represented by $-\log_{10}$ (*p*-value). The red dots represented the upregulated MZ panel and the blue dots is the downregulated MZ panel.

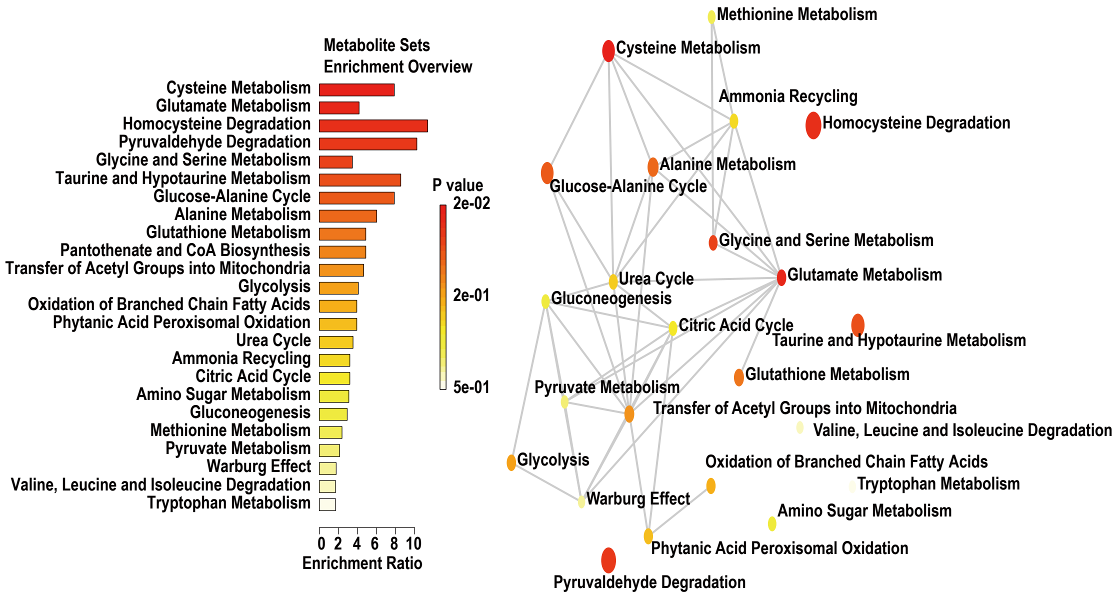

Fig. S13. Metabolic pathway analysis of PCa via MoS_2/MXene heterostructure. a) Enrichment overview and b) corresponding metabolic pathway network via MoS_2/MXene heterostructure.

Table S1. The comparisons of MoS_2/MXene with other commercial matrices.

Number	Matrix	Abbreviation	LOD[a]	S/N[b]
1	MoS_2/MXene	/	1.42 nM	23.4
2	α-Cyano-4-hydroxycinnamic acid	CHCA	168.9 nM	8.4
3	2,5-Dihydroxybenzoic acid	DHB	245.7 nM	4.3

LOD: limit of detection.

S/N: signal-noise ratio.

Table S2. Clinical information for all urine and serum biosamples.

Number	Age	PSA value	Serum biosample	Urine biosample	Type
**1**	70	6.3	√	√	BPH
**2**	59	6.2	√	√	BPH
**3**	63	8.8	√	√	BPH
**4**	71	7.9	√	√	BPH
**5**	63	5.0	√	√	BPH
**6**	66	5.2	√	√	BPH
**7**	74	5.8	√	√	BPH
**8**	58	9.8	√	×	BPH
**9**	69	9.6	√	×	BPH
**10**	68	9.7	√	√	BPH
**11**	68	8.5	√	√	BPH
**12**	78	7.4	√	√	BPH
**13**	71	9.4	√	×	BPH
**14**	67	4.7	√	√	BPH
**15**	58	7.6	√	√	BPH
**16**	67	8.4	√	√	BPH
**17**	74	6.6	√	×	BPH
**18**	67	6.8	√	√	BPH
**19**	70	7.2	√	√	BPH
**20**	70	4.1	√	√	BPH
**21**	67	6.9	√	√	BPH
**22**	71	5.1	√	√	BPH
**23**	74	5.3	√	√	BPH
**24**	70	8.2	√	√	BPH
**25**	68	10.6	√	√	BPH
**26**	57	7.3	√	√	BPH
**27**	66	4.5	√	√	BPH
**28**	75	10.7	√	√	BPH
**29**	62	4.1	√	√	BPH
**30**	64	7.4	√	√	BPH
**31**	64	8.9	√	√	BPH
**32**	73	10.6	√	×	BPH
**33**	70	5.2	√	√	BPH
**34**	67	7.3	√	×	BPH
**35**	59	4.2	√	×	BPH
**36**	73	5.6	√	×	BPH
**37**	54	8.3	√	√	BPH
**38**	67	6.3	√	√	BPH
**39**	65	5.1	√	√	BPH

**40**	61	5.6	√	√	BPH
**41**	56	5.1	√	√	BPH
**42**	63	5.4	√	√	BPH
**43**	70	6.8	√	√	BPH
**44**	66	5.1	√	×	BPH
**45**	64	9.4	√	√	BPH
**46**	61	5.0	√	×	BPH
**47**	60	5.0	√	√	BPH
**48**	70	5.1	√	√	BPH
**49**	55	7.9	√	√	BPH
**50**	67	6.6	√	√	BPH
**51**	72	7.5	√	√	BPH
**52**	72	10.6	√	√	BPH
**53**	64	4.8	√	√	BPH
**54**	68	9.7	√	√	BPH
**55**	67	10.7	√	√	BPH
**56**	64	9.8	√	√	BPH
**57**	61	9.4	√	√	BPH
**58**	56	6.9	√	√	BPH
**59**	66	7.8	√	√	BPH
**60**	63	5.9	√	√	BPH
**61**	70	9.7	√	×	BPH
**62**	63	5.3	√	√	BPH
**63**	72	9.7	√	√	BPH
**64**	70	6.3	√	√	BPH
**65**	67	7.2	√	√	BPH
**66**	69	6.8	√	×	BPH
**67**	64	4.0	√	×	BPH
**68**	67	4.8	√	√	BPH
**69**	59	8.1	√	√	BPH
**70**	54	4.6	√	√	BPH
**71**	64	5.7	√	√	BPH
**72**	67	9.6	√	√	BPH
**73**	66	7.8	√	√	BPH
**74**	55	7.0	√	√	BPH
**75**	72	5.7	√	×	BPH
**76**	62	5.6	√	×	BPH
**77**	69	4.8	√	√	BPH
**78**	74	8.7	√	√	BPH
**79**	66	10.0	√	√	BPH
**80**	77	4.9	√	√	BPH
**81**	69	7.1	√	√	BPH
**82**	67	4.6	√	√	BPH

**83**	63	8.8	√	√	BPH
**84**	67	7.4	√	√	BPH
**85**	67	7.2	√	√	BPH
**86**	70	6.7	√	√	BPH
**87**	73	5.8	√	√	BPH
**88**	57	10.8	√	√	BPH
**89**	60	4.7	√	√	BPH
**90**	64	5.7	√	√	BPH
**91**	63	6.5	√	√	BPH
**92**	68	7.3	√	√	BPH
**93**	64	9.0	√	×	BPH
**94**	59	6.9	√	√	BPH
**95**	50	7.7	√	√	BPH
**96**	70	7.4	√	√	BPH
**97**	55	8.3	√	√	BPH
**98**	70	7.0	√	√	BPH
**99**	57	5.1	√	√	BPH
**100**	68	10.1	√	√	BPH
**101**	65	6.7	√	√	BPH
**102**	67	10.8	√	×	BPH
**103**	74	6.5	√	×	BPH
**104**	72	6.4	√	×	BPH
**105**	70	9.7	√	×	BPH
**106**	67	5.8	√	√	BPH
**107**	60	6.6	√	√	BPH
**108**	50	4.1	√	√	BPH
**109**	63	7.9	√	√	BPH
**110**	69	8.5	√	×	BPH
**111**	71	6.6	√	√	BPH
**112**	73	6.4	√	√	BPH
**113**	58	9.3	√	√	BPH
**114**	70	4.7	√	√	BPH
**115**	66	8.3	√	×	BPH
**116**	65	5.0	√	√	BPH
**117**	62	5.7	√	√	BPH
**118**	57	5.0	√	×	BPH
**119**	72	9.4	√	√	BPH
**120**	68	7.9	√	×	BPH
**121**	68	6.3	√	√	PCa
**122**	75	8.2	√	√	PCa
**123**	60	7.1	√	√	PCa
**124**	69	10.1	√	√	PCa
**125**	73	10.0	√	√	PCa

**126**	74	9.7	√	√	PCa
**127**	73	7.3	√	√	PCa
**128**	74	10.2	√	√	PCa
**129**	54	5.7	√	√	PCa
**130**	70	9.5	√	√	PCa
**131**	82	4.3	√	√	PCa
**132**	67	9.2	√	×	PCa
**133**	61	4.5	√	√	PCa
**134**	63	4.9	√	√	PCa
**135**	76	9.2	√	√	PCa
**136**	74	9.2	√	√	PCa
**137**	73	5.3	√	√	PCa
**138**	81	9.6	√	√	PCa
**139**	79	9.1	√	√	PCa
**140**	49	9.2	√	√	PCa
**141**	56	9.8	√	√	PCa
**142**	67	4.7	√	√	PCa
**143**	72	9.2	√	√	PCa
**144**	72	9.5	√	√	PCa
**145**	61	8.6	√	√	PCa
**146**	63	10.6	√	√	PCa
**147**	71	5.7	√	√	PCa
**148**	68	7.6	√	√	PCa
**149**	67	8.2	√	√	PCa
**150**	64	5.7	√	√	PCa
**151**	74	9.0	√	√	PCa
**152**	52	7.2	√	√	PCa
**153**	67	9.9	√	√	PCa
**154**	73	4.4	√	√	PCa
**155**	75	5.3	√	√	PCa
**156**	68	7.2	√	√	PCa
**157**	56	10.4	√	√	PCa
**158**	67	6.6	√	√	PCa
**159**	71	7.3	√	√	PCa
**160**	64	10.4	√	√	PCa
**161**	78	4.9	√	√	PCa
**162**	73	4.6	√	√	PCa
**163**	65	5.2	√	×	PCa
**164**	63	7.9	√	×	PCa
**165**	61	9.7	√	×	PCa
**166**	75	5.2	√	√	PCa
**167**	62	7.5	√	√	PCa
**168**	59	8.1	√	√	PCa

**169**	75	7.3	√	√	PCa
**170**	60	10.3	√	√	PCa
**171**	77	10.9	√	√	PCa
**172**	77	6.5	√	×	PCa
**173**	80	6.2	√	√	PCa
**174**	71	8.2	√	×	PCa
**175**	69	4.6	√	√	PCa
**176**	59	10.1	√	√	PCa
**177**	72	5.8	√	×	PCa
**178**	72	7.7	√	×	PCa
**179**	73	8.3	√	√	PCa
**180**	61	8.8	√	√	PCa
**181**	69	10.2	√	√	PCa
**182**	79	6.2	√	√	PCa
**183**	67	8.2	√	√	PCa
**184**	63	5.3	√	√	PCa
**185**	70	4.1	√	√	PCa
**186**	78	10.0	√	√	PCa
**187**	77	8.0	√	√	PCa
**188**	71	7.8	√	√	PCa

Table S3. The comparison between the diagnostic performance between MoS_2/MXene and clinical standard technologies, including PSA and MR imaging.

**Number**	**Strategies**	**Number of biosamples**	**Sensitivity**	**Specificity**	**AUC diagnosis**	**Note**
1	Our assay (Serum)	68 PCa vs. 120 BPH	94.9%	86.2%	0.902	This work
2	Our assay (Urine)	60 PCa vs. 96 BPH	94.3%	90.2%	0.959	This work
3	Clinical standard assay (PSA)	68 PCa vs. 120 BPH	50.0%	68.3%	0.585	This work
4	Clinical standard assay (MR imaging)	68 PCa vs. 120 BPH	50.9%	65.0%	0.608	This work
